# Coffee Consumption and Oxidative Stress: A Review of Human Intervention Studies

**DOI:** 10.3390/molecules21080979

**Published:** 2016-07-28

**Authors:** Daniela Martini, Cristian Del Bo’, Michele Tassotti, Patrizia Riso, Daniele Del Rio, Furio Brighenti, Marisa Porrini

**Affiliations:** 1 LS9 Interlab Group, The Laboratory of Phytochemicals in Physiology, Department of Food Science, University of Parma, Medical School Building C, Via Volturno 39, 43125 Parma, Italy; daniela.martini@unipr.it (D.M.); michele.tassotti@studenti.unipr.it (M.T.); daniele.delrio@unipr.it (D.D.R.); furio.brighenti@unipr.it (F.B.); 2 Department of Food, Environmental and Nutritional Sciences, Division of Human Nutrition, Università degli Studi di Milano, Via G. Celoria 2, 20133 Milano, Italy; patrizia.riso@unimi.it (P.R.); marisa.porrini@unimi.it (M.P.)

**Keywords:** coffee, phenols, DNA damage, lipid damage, protein damage, antioxidant capacity, antioxidant enzymes

## Abstract

Research on the potential protective effects of coffee and its bioactives (caffeine, chlorogenic acids and diterpenes) against oxidative stress and related chronic disease risk has been increasing in the last years. The present review summarizes the main findings on the effect of coffee consumption on protection against lipid, protein and DNA damage, as well as on the modulation of antioxidant capacity and antioxidant enzymes in human studies. Twenty-six dietary intervention studies (involving acute and chronic coffee intake) have been considered. Overall, the results suggest that coffee consumption can increase glutathione levels and improve protection against DNA damage, especially following regular/repeated intake. On the contrary, the effects of coffee on plasma antioxidant capacity and antioxidant enzymes, as well as on protein and lipid damage, are unclear following both acute and chronic exposure. The high heterogeneity in terms of type of coffee, doses and duration of the studies, the lack of information on coffee and/or brew bioactive composition, as well as the choice of biomarkers and the methods used for their evaluation, may partially explain the variability observed among findings. More robust and well-controlled intervention studies are necessary for a thorough understanding of the effect of coffee on oxidative stress markers in humans.

## 1. Introduction

Coffee is one of the world’s most commonly consumed beverages, just after water and tea, probably thanks to its aromatic bouquet and its stimulating effect on the central nervous system. In 2014, coffee consumption has been estimated to reach over 50 million cups worldwide, with the highest annual consumption registered in Finland, Norway and Denmark (11.4, 8.7 and 8.0 kg per capita, respectively) [1], while per capita consumption for Italy and France was estimated in 5.6 kg and 5.4 kg, respectively [2].

Coffee is made by grinding roasted coffee beans, representing the fruit of the coffee plant, belonging to the Rubiaceae family. The main two species are *Coffea arabica* L. and *Coffea canephora*, originated in Ethiopia and in tropical Africa, respectively. These two species are traditionally used for making Arabica and Robusta coffees, with the former representing the most diffused species worldwide [1]. Even if all coffee brews could be prepared with hot water and ground coffee beans, coffee can assume a number of different forms. In Italian bars, for example, coffee is usually consumed as “espresso”, prepared by extracting finely ground powder with high-pressure hot water to produce a 30 mL serving brew. At home, coffee is instead mainly prepared with a three-part coffee-maker (called moka), in which hot water is forced up through the coffee to the top of the machine. Further options include the French press, the American-style (drip) coffee, and many others.

The use of coffee in relation to its effects on health dates back many centuries, although the first studies are linked with the Scientific Revolution. Despite the fact the association between coffee and health has been explored for many decades, the actual role of coffee drinking has been long debated, mainly because some potential negative aspects have been hypothesized. In the nineteenth century, a moderate coffee consumption was considered helpful in reducing fatigue and improving intellectual functions, but overconsumption could move coffee from food to poison. In the 1991, the International Agency for Research on Cancer (IARC) classified coffee as “possibly carcinogenic to humans“ because of a weak positive relationship between coffee consumption and the occurrence of bladder, pancreatic and ovarian cancer [3]. Recently, an international Working Group of scientists from IARC declared inadequate the evidence for the carcinogenicity of coffee drinking overall [4].

Independently from these controversies, coffee has been described as probably the most relevant source of dietary antioxidant compounds [5], which are thought to counteract the action of reactive oxygen species (ROS), the main contributors to the development of oxidative stress. Oxidative stress occurs when the cellular production of oxidant molecules exceeds the availability of antioxidants able to defeat these insults. Antioxidant-rich foods, like coffee, can therefore play an important role against this condition through the scavenging of free radicals.

Coffee contains large amounts of bioactive compounds including caffeine, phenolic compounds, trigonelline, diterpenes and soluble fiber [6]. Caffeine, representing the most widely studied psychoactive molecule in history, is a methylxanthine partially responsible of the bitter characteristics of coffee. The caffeine content in coffee may be affected by genetic and environmental factors, for instance *C. canephora* has double the content of *C. arabica* (1.5–2.5 g and 0.9–1.3 g/100 g dry matter in green seeds, respectively) [6]. The content of caffeine in a serving is also highly variable, depending on the type of roasting (e.g., light, medium or dark), coffee-making method, and extraction (e.g., regular or over extraction), with values ranging from 50 up to over 300 mg per cup [7]. Once ingested, caffeine is rapidly absorbed in the upper gastrointestinal tract, with a peak value within 60 min of ingestion [8]. The intake of caffeine has been associated with a high number of biological effects, mainly concerning the stimulation of the central and sympathetic nervous system, typically associated to a feeling of alertness after coffee consumption [9,10,11,12]. Recently, the intake of caffeine has been also reported to exert ergogenic effects, as in 2011, the request of a health claim for caffeine in this direction has received a positive opinion from the European Food Safety Authority (EFSA) [13].

Regarding diterpenes, coffee contains cafestol and kahweol, which have been found in higher amount (up to 1.2 g/100 g dry matter) in *C. arabica* seeds. Being poorly soluble in water, these compounds can be trapped by filters so they are present mainly in unfiltered coffee, as well as in espresso. In spite of the anticarcinogenic and hepatoprotective properties that have been reported in in vitro and animal models, a high intake of cafestol and kahweol seems to increase the risk of CHD, mainly through an increase of plasma LDL. A meta-analysis on 11 trials showed an increase of 5.0 mg/dL and 0.9 mg/dL in serum total cholesterol with each 10 mg of cafestol or kahweol consumed per day for four weeks, respectively, with a linear effect up to 100 mg of cafestol/day [14].

**Figure 1:**
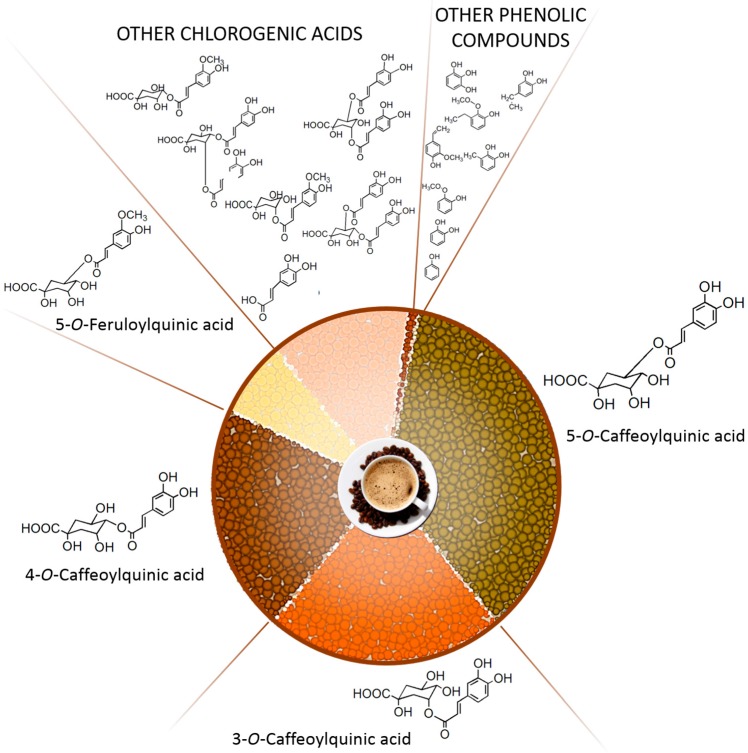
Phenolic compounds present in coffee.

Among phenolic compounds, chlorogenic acids (CGAs) are the most abundant in coffee, representing more than 98% of its total phenolic content, as shown in Figure 1, while the remaining 2% is composed of alkylmethoxyphenols, alkylphenols, methoxyphenols, and other phenolics such as pyrogallol, catechol, and phenol [15].

CGAs are a group of esters formed by hydroxycinnamic acids, such as caffeic acid, ferulic acid and *p*-coumaric acid, bound to quinic acid in a range of conjugated structures known as caffeoylquinic acids, feruloylquinic acids, and *p*-coumaroylquinic acids [16]. The isomers of these compounds depend on the site of esterification that can occur at positions 1, 3, 4 or 5 of the quinic acid moiety [17]. The most abundant and most studied CGA is 5-caffeoylquinic acid (5-CQA), accounting for about 50% of the total content in green coffee beans [15]. As reported for caffeine and diterpenes, the content of CGAs in coffee may vary depending on several factors. For example, genetics has been shown to deeply influence both the occurrence of CGAs, with *C. robusta* generally displaying a higher CGA content than *C. arabica* [18,19], and the proportion of individual CGA subgroups. Environmental factors, like rainfall level and different mean temperatures, have been reported to affect coffee CGA content even in the same cultivar grown in the same growing area over different years [20].

As regards the processing of coffee, roasting appears to be a critical process for the evolution of flavor, aroma and color in traditional coffee beverages as well as for their CGA content, with a relevant loss of CQAs (up to 90%) in dark roasted beans compared to unroasted green beans. In addition, CGA levels may be affected by the brewing method so that an over-extraction (~55 mL) has been reported to increase over 85% the total CGAs with respect to the regular extraction (~23 mL) [7]. However, CGAs are generally preserved during coffee brewing, resulting in a final concentration that could be higher than 150 mg per serving [7]. Therefore, in spite of the huge CGA loss during roasting and the differences among preparations, coffee still remains the major dietary source of CGAs, to a level that regular coffee intake by heavy drinkers may lead to a daily intake higher that 1 g.

Despite the high amounts of ingested CGAs, only low concentrations of chlorogenic acids in their native forms have been found in blood, and extensive metabolic transformations were previously described [21]. Actually, if an extremely low amount of these compounds is absorbed in the small intestine, most of CGAs reach the colon, where they can be metabolized by the local microbiota. Metabolites are then absorbed, further conjugated in the liver, and distributed to the tissues [17]. At least ten conjugates, dihydroisoferulic acid 3-*O*-glucuronide, caffeic acid 3-sulfate, as well as the sulfate and glucuronide derivatives of 3,4-dihydroxyphenylpropionic acid, were identified in human plasma and/or urine after coffee consumption [22].

## 2. Objective and Literature Search Strategy

The present review aims to summarize the main findings of human intervention studies investigating the effects of coffee consumption on oxidative stress. In detail, the effects of coffee consumption on markers of lipid, protein and DNA damage, as well as on markers related to antioxidant capacity and antioxidant enzymes, are reviewed. Most of these markers are considered sensitive and specific biomarkers for antioxidant status and can be useful for a better comprehension of the role of antioxidant-rich foods, including coffee, against oxidative stress and related conditions.

**Figure 2:**
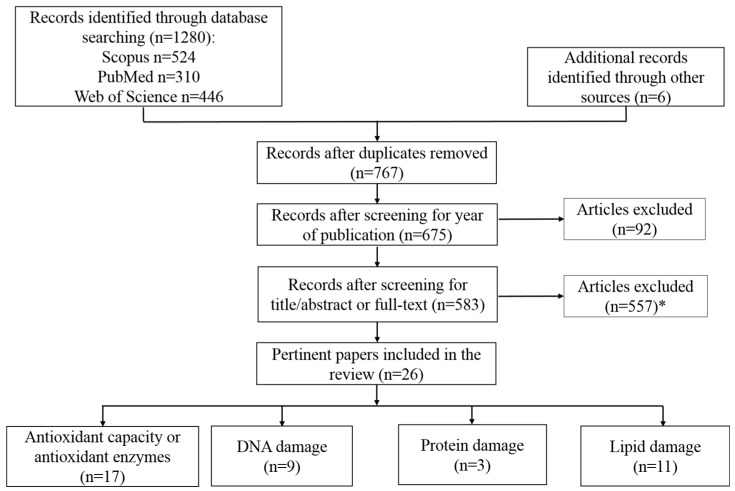
Flow diagram of article selection. * Studies were excluded for the following reasons: (1) not in English; (2) did not concern intervention studies; (3) not including in vivo markers of oxidative stress.

PUBMED, Web of Science and Scopus databases were searched to identify pertinent articles. The searches used the combination of the following terms: “coffee”, “antioxidant capacity”, “antioxidant activity”, “DNA damage”, “protein damage”, “lipid damage”, “oxidation” and “human”. Reference lists of the retrieved papers were also searched for additional articles. The search strategy is summarized in Figure 2.

A total of 26 pertinent human intervention studies were identified, published in 18 different peer-reviewed journals and conducted in 12 different countries, mainly Austria, Germany and Italy. More than half of the 26 studies were published during the last 5 years, suggesting a growing interest for this topic. The results obtained are reported in Table 1 and Table 2 describing the type of coffee used in the investigation, with details about the way of preparation and the content of bioactive compounds in the final brew, when available. Moreover, the duration of the intervention, the number of subjects and their characteristics, the use of a control/placebo food, the selected markers and the main findings are reported.

Three out of the 26 studies investigated the effects of both acute and chronic interventions with coffee, while 8 were only acute studies (i.e., single dose), and 15 were chronic (medium-long term) dietary intervention.

## 3. Results

### 3.1. Total Plasma Antioxidant Capacity and Antioxidant Enzymes

The effect of coffee consumption on the modulation of plasma antioxidant capacity was evaluated in 10 of the selected studies. Two studies reported both *acute* and *chronic* interventions [23,24], four were acute studies [25,26,27,28], while four were chronic intervention studies [34,35,36,37]. Three studies were not placebo-controlled [24,34,37] and/or did not provide information about the bioactive composition of coffee [24,26,27]. Total radical trapping antioxidant power (TRAP), Trolox-equivalent antioxidant capacity (TEAC), total antioxidant status (TAS) and oxygen radical absorbance capacity (ORAC) emerged as the most used methods. They differ from each other in terms of reaction mechanisms, oxidant and target/probe species, reaction conditions and expression of the obtained results.

Only four (three acute and one chronic intervention) out of 10 studies reported a significant increase in total plasma antioxidant capacity following coffee consumption. In particular, Moura-Nunes and colleagues documented that a single serving of 200 mL of coffee increased plasma antioxidant capacity, determined through FRAP and TRAP assays, by 2.6% and 7.6%, respectively, in a group of healthy subjects [27]. Natella and coworkers observed an increase in plasma antioxidant capacity (measured as TRAP) and thiol (SH) groups after consumption of 200 mL of coffee, which was even more pronounced than that observed with the same amount of tea (+6% versus +4%, respectively) [28]. Agudelo-Ochoa et al. [23] reported that a single serving of 400 mL of coffee, providing either 420 mg or 780 mg of chlorogenic acid, significantly increased plasma antioxidant capacity (+6% and +5%, respectively) in a group of healthy volunteers, but these effects were lost following a long term intervention. Finally, Corrêa et al. [34] reported that a 4-week intake of 150 mL/day of medium light roast (MLR) or medium roast (MR) paper-filtered coffee increased the levels of TAS by about 21% and 26% respectively, while ORAC increased only after the consumption of medium light roast paper-filtered coffee. The lack of homogeneous results could be due to differences in the duration of the intervention, the type and the amount of coffee provided, the composition in bioactive compounds, and the method used for the determination of antioxidant capacity.

The effect of coffee in the modulation of endogenous antioxidant enzymes has been assessed in seven chronic intervention trials. The duration of the studies varied from 1 to 4 weeks. Superoxide dismutase (SOD), catalase (CAT), glutathione peroxidase (GPx), glutathione reductase (GSR) and glutathione S-transferases (GSTs) were the most widely studied enzymes.

Overall, the results obtained are conflicting and do not provide evidence about the role of coffee in the modulation of antioxidant enzymes. For example, Corrêa et al. [34] reported an increase in erythrocyte antioxidant enzyme activity in healthy subjects following a 4-week intervention with 150 mL/day of MLR and MR paper-filtered coffee. In particular, SOD activity increased by 52% and 75% in MLR and MR group, while GPx activity by 62% and 49%, respectively. Moreover, both the interventions significantly increased CAT activity by about 13%. Kotyczka et al. [38] documented that a 4-week intake of light dark roasted coffee (rich in chlorogenic acid) resulted in increased erythrocyte activities of SOD, GPx and CAT by 12%, 25% and 22%, respectively. On the contrary, the intake of dark roasted coffee (poor in chlorogenic acid) decreased erythrocytes SOD and GPx activity by 5.8% and 15%, respectively. Misik and colleagues showed that a 5-day coffee intake (800 mL/day) did not significantly affect SOD and GPx activity in the cytosolic fractions of the lymphocytes of healthy volunteers [36].

The activity of GST and GSR was evaluated in two studies. Steinkellner and colleagues reported that a 5-day intervention with 1 L unfiltered coffee/day increased GST activity in plasma, but not in saliva, in a group of healthy individuals [39]. Bakuradze et al. [40] showed a significant increase in GSR blood level activity following 4-week consumption of 750 mL/day of freshly brewed coffee.

Eight studies (seven chronic interventions and one acute trial) also investigated the role of coffee in the modulation of blood glutathione (GSH) levels as a substrate of GPx and GST enzymes. Four out of seven chronic intervention studies documented an increase in GSH levels [38,40,41,42], while two long-term studies [35,36] and one study performing both an acute and a chronic intervention [24] did not show any significant effect. Misik and coworkers attributed the lack of effects to the degradation and metabolic conversion of different coffee constituents in the body [36], while for Teekachunhatean and colleagues the short duration of the intervention could be at least partially responsible for the absence of a relevant effect [24].

### 3.2. Protein Damage

The effect exerted by coffee intake on protein damage has been investigated only in three studies [35,36,43]. Those studies differed for the fed coffee type, which was instant coffee [35], coffee homemade with participants’ coffee machines [36] or coffee prepared by paper filtration [43]. In two studies [35,43], partially performed by the same research group, a controlled intervention trial with a cross-over design was scheduled, where subjects were randomized to consume coffee or a control drink (water) for periods of 5 days each, spaced out by a washout phase and dietary restriction. Differently, Kempf and coworkers investigated the effect of two different doses of coffee (4 and 8 cups, corresponding to 600 and 1200 mL respectively) by using a simple experimental design: subjects had to follow 4 weeks of restrictive diet, followed by 4 weeks with 4 cups coffee/day and 4 weeks of 8 cups coffee/day [43]. The studies performed by Hoelzl et al. [35] and Misik et al. [36] were also similar for what concerns the volunteers, being nonsmokers and normal weight young adults in both cases, contrarily to the study by Kempf et al. [43] where subjects with a high risk of type 2 diabetes were recruited. In all the three studies, the marker of protein damage was 3-nytrotirosine (3-NT), a stable post-translational modification in proteins, deriving from the reaction of free or protein-bound tyrosine with reactive nitrogen oxide species including peroxynitrite, nitrogen dioxide and nitrous acid. 3-NT has been suggested to be associated with coronary heart disease (CAD) independently of traditional risk factors [49]. The marker was monitored by LC-MS/MS in two studies [35,36] and by an enzyme immunoassay in the third one [38]. A significant effect of coffee was found only by Hoelzl and colleagues, who observed a significant decrease of 3-NT (16.1%) after 4-week coffee intake [35]. Despite many similarities between the investigations by Hoelzl et al. [35] and Misik et al. [36] (i.e., duration of the intervention, amounts of CGA provided), the latter found no significant effect after coffee intake, in line with the observations of Kempf and colleagues [43]. A possible explanation for such different findings might be linked to the amount of coffee bioactives other than CGA provided with the two different coffee brews. However, Hoelzl et al. [35] provided information only about CGA, making a clear comparison between the two investigations practically impossible.

The small number of investigations about the possible role of coffee consumption on markers of protein damage, together with the heterogeneity of the findings, calls for further studies focusing on this aspect of oxidative damage to biomolecules.

### 3.3. Lipid Damage

The effect of coffee consumption on markers of lipid oxidation has been investigated in 12 studies [24,25,26,29,30,31,32,34,35,36,43,44]. Five out of 12 studies investigated only the acute effect of coffee consumption [25,26,29,30,31], five were chronic intervention studies [34,35,36,43,44], while two studies investigated both acute and chronic effects [24,32].

In these studies, isoprostanes (IsoPs) and malondialdehyde (MDA) were the most frequently considered markers of lipid damage. In detail, isoprostanes are a class of end-products of peroxidation of arachidonic acid, which are collectively referred as F2-IsoPS due to their similarity to prostaglandin F2α. Among them, 8-isoprostaglandin F2α (8-Iso PGF2α) is commonly used for evaluating oxidative stress, through both chromatographic techniques and immunoassays. MDA is instead a three carbon, low molecular weight aldehyde representing the main product of polyunsaturated fatty acid peroxidation. It is characterized by a high toxicity due to its ability to react with other molecules like DNA and protein [50]. In all the studies evaluating MDA, the reaction with 2-thiobarbituric acid (TBA) was used, so results were reported as TBA reacting substances (TBARS) instead of MDA. In spite of the risk of overestimation of MDA, the TBARS method represents the most common test for evaluating lipid peroxidation.

Besides 8-IsoPGF2 and MDA, further markers of lipid damage and/or protection considered in the present review were oxidized LDL, resistance to LDL oxidation, serum LDL-conjugated dienes and hydroxyl fatty acids. The analysis of the main findings revealed that most of the interventions failed to demonstrate a significant decrease in markers of lipid damage with exception of results found by Ochiai et al. [30] and Sirota et al. [31]. The former reported a significantly reduced urinary 8-epiPGF2α following consumption of a coffee beverage (providing 600 mg of CGAs) when compared with placebo in healthy men. Results showed that consumption of 200 mL Turkish roasted coffee during a meal based on red-meat cutlets resulted in a significant inhibition of postprandial plasma MDA. No effect between treatments and control/placebo were instead found by other authors [25,29,32]. The investigation by Leelarungrayub et al. [26] deserves a special mention, because it reports a significant higher level of MDA in men consuming caffeinated coffee, when compared to decaffeinated coffee or control, followed by a submaximal exercise test. Authors reported that, similarly to what observed in previous investigations, results demonstrated an increased intramuscular fat oxidation following consumption of caffeine-rich foods.

Among medium-long term intervention studies, a significant decrease in isoprostanes was observed only in three studies [30,35,43], while no significant change was reported by Mursu et al. [32] and Misik et al. [36]. Even if the exact composition of the coffee used has not been always provided, the differences in the findings seem to be at least partially attributable to the different composition of the brews.

For what concerns the other markers of lipid damage, only Yukawa et al. [44] found a modest reduction of LDL oxidation susceptibility and a decrease of MDA levels following consumption of 3 coffees/day for 1 week. No significant effect was instead found by Mursu et al. [32] on serum LDL-conjugated dienes and plasma hydroxyl fatty acids, or by Teekachunhatean et al. [24] on MDA levels [24] and by Hoelzl et al. [35] on both MDA and oxidized LDL.

### 3.4. DNA Damage

The role of coffee on markers of DNA damage has been investigated in nine studies (eight chronic interventions and one acute study), four of which performed by the same research group [33,40,46,47]. Eight out of nine studies measured the levels of DNA damage through the comet assay, a single cell gel electrophoresis technique widely used also in human biomonitoring and dietary intervention studies [51,52]. Three of these studies [33,46,47] investigated the effect of coffee on spontaneous DNA strand breaks (SBs), which directly reflect the background DNA oxidative damage within cells. Background SBs may derive from endogenous and/or exogenous exposure to DNA damaging agents and/or may reflect incomplete DNA repair. The consumption of coffee was associated to reduce DNA SBs in healthy volunteers. Bakuradze et al. [33] reported that the ingestion of 800 mL of coffee (200 mL every 2 h) significantly reduced (up to 30%) DNA SBs in a short-term human intervention study. The same research group documented that coffee consumption (3 × 250 mL per day) was associated with DNA-protective effects (*p* < 0.001) in a 4-week, double-blind, randomized, crossover intervention [46]. Finally, the same authors showed that a daily consumption of 750 mL of fresh dark roast coffee decreased by 27% spontaneous DNA SBs in a 4-week, randomized, controlled trial [47]. 

Five out of eight studies investigated the effects of coffee consumption on oxidized DNA bases through the exploitation of specific enzymes such as formamidopyrimidine-DNA glycosylase (FPG) and/or endonuclease III (Endo III), able to detect oxidized purines and pyrimidine bases, respectively [53,54]. Some studies also evaluated the resistance to oxidatively induced DNA damage, using H_2_O_2_ [35,36,48], BPDE [39] and Trp-P-2 [47] as oxidative agents.

Bichler and coworkers showed that the consumption of 600 mL coffee (400 mL paper filtered and 200 mL metal filtered/day) for 5 days reduced both endogenous (FPG and Endo III-sensitive sites by 64% and 48%, respectively) and oxidatively induced DNA damage (measured as DNA resistance to H_2_O_2_ and Trp-P-2 by 17% and 35%, respectively) in a group of healthy volunteers [47]. Steinkellner and colleagues documented that a 5-day intervention with 1 L unfiltered coffee/day increased cell protection from (±)-anti-B[*a*]P-7,8-dihydrodiol-9,10-epoxide oxidative insult in a group of healthy subjects [39]. Misik et al. [36] reported a significant reduction in the levels of FPG (by 12.3%) and Endo III-sensitive sites (by 10%), but not DNA resistance to H_2_O_2_-oxidative treatment, following the administration for 5 days of 800 mL/day of paper filtered coffee. Hoelzl and colleagues showed that the intake of a comparable amount of instant coffee co-extracted from green and roasted beans did not significantly affect the levels of FPG and Endo III-sensitive sites, and H_2_O_2_-induced DNA damage, in a group of healthy individuals [35].

Another marker widely used to measure oxidized base lesion is 8-oxo-2′-deoxyguanosine (8-OHdG), as, among all purine and pyridine bases, guanine is the most prone to oxidation and a common biomarker reflecting the balance between oxidative damage and repair rate [55]. The role of coffee in the modulation of 8-OH-dG was evaluated only in one study, with positive results [48]. The study was performed in a group of patients affected by chronic hepatitis C and the authors documented that 8-OHdG levels were significantly lower during coffee intake (30-day consumption of 4 cups of coffee/day), with almost a three-fold decrease.

## 4. Conclusions

During the last five years, coffee has been the objective of several studies for its potential role in human health, with a specific focus on the prevention of several chronic degenerative diseases. The current review summarized the main findings of 26 studies performed in humans, with the aim of comparing results on the effect of coffee consumption on the main markers of oxidative damage to lipid, DNA and protein, as well as on the modulation of antioxidant capacity and antioxidant enzymes in humans. Studies were performed on healthy subjects with the exception of one study in which patients with chronic hepatitis C were recruited.

Overall, a high heterogeneity among studies was observed, mainly in terms of doses and duration of the interventions, and, in several studies, information concerning the polyphenol content of the coffee used was lacking. Only a few studies provided the content of CGAs, caffeine and other bioactive compounds in the fed coffee brew, and, in general, they did not describe in great detail the way coffee was prepared (i.e., grams of coffee used for each dose). This lack of information about the composition of the brews makes the comparison among studies extremely difficult, with an objective evaluation of a dose-response effect almost impossible. Therefore, the need for more detailed information about the chemical composition of coffees in future studies appears crucial for a more accurate analysis of results.

Despite these complications, the main findings of the reviewed works seem to suggest that consumption of coffee may increase glutathione levels and reduce the levels of DNA damage. These effects are more evident in chronic interventions than in acute studies, letting hypothesize that a long-term exposure to coffee and/or its bioactive compounds is needed to obtain such putative health effects. On the contrary, an extreme heterogeneity of the results has been observed for total plasma and serum antioxidant status, as well as for protein and lipid damage. This could be attributed to the different biomarkers and methods used for their evaluation. Based on the difficulties described above, a comprehensive understanding of the beneficial effects of coffee on oxidative stress markers, through the development of robust and well-controlled intervention studies, is required.

## Figures and Tables

**Table 1:** Role of coffee in the modulation of oxidative stress biomarkers: overview of the acute human intervention studies.

Reference	Subjects	Type of Coffee and Composition	Doses	Study Design	Markers			
					Antioxidant Capacity/Enzymes	Lipid Damage	DNA Damage	Protein Damage
Agudelo-Ochoa et al. [23]	74 healthy subjects (38 males, 37 females), mean age of 38.5 ± 6.9 years, mean BMI 24.1 ± 2.6 kg/m^2^ Control group: 13 males, 12 females; 14 subjects aged 20–40 years, 11 aged 41–60 years Group MCCGA: 12 males, 13 females; 14 subjects aged 20–40 years, 11 aged 41–60 years Group HCCGA: 12 males, 12 females; 10 subjects aged 20–40 years, 14 aged 41–60 years	Coffee 1 (MCCGA): Colombian Arabica coffee Composition: total CGAs 105 ± 4.1 mg/100 mL, cafestol 0.19 ± 0.03 mg/100 mL, kahweol 0.22 ± 0.03 mg/100 mL, caffeine 47 ± 1.4 mg/100 mL Coffee 2 (HCCGA): Colombian Arabica coffee Composition: total CGAs 195 ± 6.9 mg/100 mL, cafestol 0.19 ± 0.01 mg/100 mL, kahweol 0.23 ± 0.02 mg/100 mL, caffeine 49 ± 1 mg/100 mL	Coffee group 1: 400 mL of MCCGA coffee Coffee group 2: 400 mL of HCCGA coffee Control group: no coffee	Parallel intervention	↑AC (FRAP)			
Teekachunhatean et al. [24]	11 healthy men, (mean age 21.09 ± 7.97 years, mean BMI 20.80 ± 2.27 kg/m^2^)	Coffee 1: Coffee enema, prepared mixing 4 g of ground coffee beans with 100 mL of purified water. Composition: n.d Coffee 2: Coffee for oral procedure: ready-to-drink coffee beverage Composition: n.d	Coffee group 1: Coffee enema (500 mL) Coffee group 2: 180 mL ready-to-drink coffee Control group: n.d	Randomized, two-phase, crossover intervention	=GSH ↓TAC	=MDA		
Bloomer et al. [25]	16 healthy subjects (8 males, 8 females; mean age 29.2 ± 14.4 years, mean BMI 23.3 ± 2.2 kg/m^2^)	Coffee: caffeinated and decaffeinate Composition: 175 mg caffeine (caffeinated), 15 mg caffeine (decaffeinated) per 16 ounces	Coffee group 1: 16 ounces of freshly brewed caffeinated coffee following milk shake consumption Coffee group 2: 16 ounces of freshly brewed decaffeinated coffee following milk shake consumption Control group: 16 ounces of bottled water, following milk shake consumption	Parallel intervention	=TAC	=MDA		
Leelarungrayub et al. [26]	26 sedentary men Group 1 (Caffeine): 10 males, mean age 20.5 ± 0.53 years, mean BMI 22.84 ± 2.65 kg/m^2^ Group 2 (Decaffeinated): 10 males, mean age 20.3 ± 0.48 years, mean BMI 22.27 ± 3.56 kg/m^2^ Group 3 (Control): 6 males, mean age 20.17 ± 0.98 years, mean BMI 23.06 ± 3.60 kg/m^2^	Coffee 1: Caffeinated coffee Composition: n.d. Coffee 2: Decaffeinated coffee (Instant freeze dried) Composition: n.d.	Coffee group 1: Caffeinated (5 mg caffeine/kg bw) coffee followed by a submaximal exercise test Coffee group 2: decaffeinated coffee followed by a submaximal exercise test Control group: No coffee consumption followed by a submaximal exercise test	Parallel intervention	=TAC	↑MDA		
Moura-Nunes et al. [27]	10 subject (3 males and 7 females), range age 22–57 years, BMI n.d.	Coffee: Instant coffee 100% Arabica prepared dissolving 8 g in 200 mL boiling water. Composition: n.d.	Coffee group: 200 mL instant coffee beverage Control group: 200 mL water	Randomized, controlled, crossover intervention	↑AC (TRAP and FRAP)			
Natella et al. [28]	10 healthy nonsmoker subjects (5 males, 5 females), age and BMI n.d.	Coffee: Coffee Lavazza Qualità Rossa Composition: caffeine 181 ± 10 mg/cup, theobromine 28.9 ± 1.1 mg/cup, total phenols 161 ± 9 mg of GAE/cup, TRAP 10.1 ± 0.6 mM ROO^·^eq./cup Tea: Twining Earl Gray Composition: caffeine 130 ± 7 mg/cup, theobromine 5.9 ± 0.4 mg/cup, total phenols 87 ± 9 mg of GAE/cup, TRAP 1.3 ± 0.1 mM ROO·equiv/cup	Coffee group: 200 mL of brewed coffee Control group: 200 mL of Twining Earl Gray tea	Baseline and post-intervention	↑AC (TRAP) ↑SH groups (ns)			
Ochiai et al. [29]	14 healthy men, (mean age 36.2 ± 7.8 years, mean BMI 22.7 ± 1.8 kg/m^2^)	Coffee: Coffee polyphenol (CPP) prepared from green coffee beans by hot water extraction Composition: Total CQA content 80.7%	Coffee group: CPP (600 mg CGAs, co-administered with the glucose solution) Control group: 225 mL of a 75-g Glu-equivalent test solution	Single-blind, randomized, controlled, crossover intervention		↑MDA (no differences among treatment) ↑IsoPs (no differences among treatment)		
Ochiai et al. [30]	13 healthy men, (mean age 44.9 ± 1.4 years, mean BMI 21.9 ± 0.6 kg/m^2^)	Coffee: Coffee bean polyphenol (CBP) beverage Composition: 600 mg CGA/100 mL water	Coffee group: 600 mg CGAs (equivalent to two cups of coffee) in 100 mL of water after a test meal Control group: 100 mL of water after a test meal	Double-blind, randomized, crossover intervention		↑MDA (no differences among treatment) ↓IsoPs		
Sirota et al. [31]	10 healthy subjects Characteristics of the subjects: n.d.	Coffee 1: Turkish roasted ground coffee (A) Composition: 110 mg polyphenols/g∙dm Coffee 2: Turkish roasted ground coffee (AG) enriched by 2% freeze-dried powder of green beans Composition: 123 mg polyphenols/g∙dm	200 mL coffee A, AG or water together with 250 g red-meat cutlets	Crossover intervention		↓MDA concentration ↓MDA absorption after coffee A, less after coffee AG		
Mursu et al. [32]	45 nonsmoking volunteer men (mean age, 26 ± 6 years and BMI < 32 kg/m^2^). Only 35 subjects completed the trial	Coffee: Finely ground coffee, repared by filtering through paper (7–8 g of grounds per one 150-mL cup) Composition: 80.9 ± 3.3 mg/100 mL of phenolic acids, with CGA as major compound (~90%)	Coffee group: 1–2 cups (150–300 mL, respectively) Control group: No coffee	Parallel intervention		=LDL-conjugated dienes =Plasma hydroxy fatty acids =F2-IsoPs		
Bakuradze et al. [33]	13 healthy men subjects (mean age 23 ± 2.4 years, mean BMI 23.8 ± 1.6 kg/m^2^)	Arabica coffee, freshly prepared in a pad machine Composition: 16.7 ± 0.7 mg/g caffeine, 10.4 ± 0.9 mg/g CGA, 1.1 ± 0.2 NMP and 3.9 ± 0.3 mg/g trigonelline	Coffee group: 200 mL Control group: n.d	Baseline and post intervention			↓SBs	

AC: antioxidant capacity; BMI: body mass index; CGA: chlorogenic acids; FRAP: ferric-reducing antioxidant power; GAE: gallic acid equivalent; GSH: reduced glutathione; IsoPs: isoprostanes; MDA: malondialdehyde; NMP: *N*-methylpyridinium; SBs: strand breaks; TAC: total antioxidant capacity; TRAP: total reactive antioxidant potential.

**Table 2:** Role of coffee in the modulation of oxidative stress biomarkers: overview of the long-term human intervention studies.

Reference	Subjects	Type of Coffee and Composition	Doses	Study Design	Markers			
					Antioxidant Capacity/Enzymes	Lipid Damage	DNA Damage	Protein Damage
Agudelo-Ochoa et al. [23]	74 healthy subjects (38 males, 37 females), mean age of 38.5 ± 6 9 years, mean BMI 24.1 ± 2.6 kg/m^2^ Control group: 13 males, 12 females; 14 subjects aged 20–40 years, 11 aged 41–60 years Group MCCGA: 12 males, 13 females; 14 subjects aged 20–40 years, 11 aged 41–60 years Group HCCGA: 12 males, 12 females; 10 subjects aged 20–40 years, 14 aged 41–60 years	Coffee 1 (MCCGA): Colombian Arabica coffee Composition: total CGAs 105 ± 4.1 mg/100 mL, cafestol 0.19 ± 0.03 mg/100 mL, kahweol 0.22 ± 0.03 mg/100 mL, caffeine 47 ± 1.4 mg/100 mL Coffee 2 (HCCGA): Colombian Arabica coffee Composition: total CGAs 195 ± 6.9 mg/100 mL, cafestol 0.19 ± 0.01 mg/100 mL, kahweol 0.23 ± 0.02 mg/100 mL, caffeine 49 ± 1 mg/100 mL	Coffee group 1: 400 mL/day of MCCGA for 8 weeks Coffee group 2: 400 mL/day of HCCGA for 8 weeks Control group*:* no coffee for 8 weeks	Parallel intervention	↓AC (FRAP)			
Teekachunhatean et al. [24]	11 healthy men, mean age 21.09 ± 7.97 years, mean BMI 20.80 ± 2.27 kg/m^2^	Coffee 1: (Enema, coffee prepared mixing 4 g of ground coffee beans with 100 mL of purified water. Composition: n.d. Coffee 2: coffee for oral procedure: ready-to-drink coffee beverage Composition: n.d.	Coffee group 1: Coffee enema (500 mL, 3 times/week for 6 visits) Coffee group 2: 180 mL ready-to-drink coffee (2/day for 11 days) Control group: n.d.	Randomized, crossover intervention	=GSH ↓TEAC	=MDA		
Mursu et al. [32]	45 nonsmoking men (mean age, 26 ± 6 years and BMI < 32 kg/m^2^). 43 subjects completed the trial	Coffee: finely ground coffee, repared by filtering through paper (7–8 g of grounds per one 150-mL cup) Composition: 80.9 ± 3.3 mg/100 mL of phenolic acids, with CGA as major compound (~90%)	Coffee group 1: 3 cups (450 mL/day) of coffee for 3 weeks Coffee group 2: 6 cups (900 mL/day) of coffee for 3 weeks Control group: No coffee consumption for 3 weeks	Parallel intervention	=GPx	=Serum LDL-conjugated dienes =Plasma hydroxy fatty acids =F2-IsoPs		
Corrêa et al. [34]	Twenty healthy subjects (6 males, 14 females), mean age 49 ± 9 years, BMI n.d.	Coffee 1: MLR-Medium Light Roast paper-filtered coffee. 15 g per one 150-mL cup Composition: total phenolic content 11.09 ± 0.29 mg 5-CQAE/mL, total CGAs 1.98 ± 0.02 mg 5-CQAE/mL, caffeine 1.54 ± 0.01 mg/mL Coffee 2: Medium Roast (MR) paper-filtered coffee. 15 g per one 150-mL cup. Composition: total phenolic content 10.53 ± 0.56 mg 5-CQAE/mL, total CGAs 1.24 ± 0.01 mg 5-CQAE/mL, caffeine 1.63 ± 0.02 mg/mL	Coffee group 1: 150 mL MLR for 4 weeks Coffee group 2: 150 mL MR for 4 weeks Control group: n.d.	Randomized, cross-over intervention	↑AC (TAS and ORAC) ↑GPx ↑CAT ↑SOD	=OxLDL =IsoPs		
Hoelzl et al. [35]	29 subjects (13 males: mean age 25.2 ± 5.6 years, mean BMI 23.0 ± 1.7 kg/m^2^; 16 females: mean age 29.3 ± 10.9 years, mean BMI 21.8 ± 2.4 kg/m^2^)	Coffee: mix of 35% green and 65% roasted coffee water extracts Composition: total CGA 8.91% dm	Coffee group: 800 mL coffee/day over 5 days Control group: 800 mL water/day over 5 days	Randomized, controlled, crossover intervention	=GSH =TAC	↓8-IsoPs =OxLDL =MDA	=EndoIII and FPG sensitive sites =H_2_O_2_-induced DNA damage	↓3NT
Misik et al. [36]	38 healthy nonsmokers subjects (14 males, 24 females), mean age 27.6 ± 8.0 years, mean BMI 22.3 ± 2.8 kg/m^2^	Coffee: coffee brand “Tchibo Beste Bohne” (100% Arabica) prepared by paper filtration. Composition: total CGA 125 mg/100 mL, caffeine 65 mg/100 mL and NMP 3.1 mg/100 mL	Coffee group: 800 mL coffee/day over 5 days Control group: 800 mL water/day over 5 days	Randomized, controlled, crossover intervention	=SOD =GPx =GSH =TAC	=IsoPs =OxLDL =MDA	↓FPG-sensitive sites ↓EndoIII sensitive sites (ns) =H_2_O_2_-induced DNA damage	=3NT
Revuelta-Iniesta & Al-Dujaili [37]	20 subjects (7 males, 13 females), mean BMI 24.23 ± 4.6 kg/m^2^, age n.d.	Coffee 1: BC (black coffee): Sainsbury’s Original Blend Cafetière Coffee Composition: polyphenols ranging from 1451 mg GAE/100 mL (Filter method) to 2475 mg GAE/100 mL (French Cafetiere) Coffee 2: GC (green coffee): Ethiopian Harrar 4 (100% Arabica) Composition: polyphenols ranging from 972 mg GAE/100 mL (French Cafetiere) to 2052 mg GAE/100 mL (Italian Cafetiere)	Coffee group 1: 40 g/day of GC for 2 weeks Coffee group 2: 40 g/day of BC for 2 weeks Control group: n.d.	Randomized, cross-over intervention	=AC (FRAP)			
Kotyczka et al. [38]	30 healthy subjects, mean age 26 ± 1 years, mean BMI 23.2 ± 0.5 kg/m^2^	Coffee 1: CBs 30 g of roast powder. Dark roast coffee beverage (NMP-CB, 260 °C, 5 min) Composition: rich in NMP (785 μmol/L) and low in CGA (523 μmol/L). Coffee 2: Light roast coffee beverage (CGA-CB, 260 °C, 2 min) Composition: rich in CGA (4538 μmol/L) and low in NMP (56 μmol/L)	Coffee group 1: 500 mL/day of light roast coffee for 4 weeks Coffee group 2: 500 mL/day of dark roast coffee for 4 weeks Control group: n.d.	Randomized, longitudinally, intervention	↑SOD (CGA-CB) ↓SOD (NMP-CB) ↑CAT ↑GPx (CGA-CB) ↓GPx (NMP-CB) ↑tGSH (CGA-CB) ↑tGSH (NMP-CB)			
Steinkellner et al. [39]	First trial: 10 healthy nonsmokers subjects (3 males, 7 females), mean age 26 ± 4 years, mean bw 75 ± 9 kg Second trial: 14 subjects, mean age 25 ± 6 years, mean bw 74 ± 10 kg Third trial: subjects (number n.d.), mean age 26 ± 6 years, mean bw 72 ± 8 kg	Coffee 1: unfiltered coffee: Ground coffee (“Brasil sanft”) boiled in 10.0 L tap water for 5 min and pressed through a metal mesh Composition: n.d. Coffee 2: filtered coffee Composition: n.d.	*First trial:* Coffee group: 7 cups/day (in total 1 L) of unfiltered coffee over 5 days Control group*:* n.d. *Second trial:* Coffee group 1: 7 cups/day (in total 1 L) of unfiltered coffee for 3 days Coffee group 2: 7 cups/day (in total 1 L) of filtered coffee for 3 days Control group: n.d. *Third trial:* Coffee group: 7 cups/day (in total 1 L) of unfiltered coffee for 5 days Control group: n.d.	First trial: Baseline and post-intervention Second trial: Parallel intervention Third trial: Baseline and post-intervention	=GST in saliva ↑GST in plasma		↓BPDE-induced DNA damage	
Bakuradze et al. [40]	33 healthy males (range age 20–44 years; mean BMI 25.6 ± 3.7 kg/m^2^)	Coffee: special roasted and blended Arabica coffee rich in both green and roast bean constituents, especially in CGA and NMP Composition: 72 mg/L NMP, 263.6 mg/L trigonelline, 720 mg/L caffeine	Coffee group: 750 mL/day (in three equal portions) for 4 weeks Control group: 750 mL/day water for 4 weeks	Randomized, controlled, cross-over intervention	↑GSH =GSSG ↑GSR activity		↓SBs ↓ FPG-sensitive sites	
Esposito et al. [41]	23 healthy subjects (18 treated and 5 controls), smokers and non smokers Coffee group: 7 males, 11 females; age range 19–25 years, mean BMI M 24.7 ± 2.9 kg/m^2^, F 22.8 ± 5.4 kg/m^2^ Control group: 2 males, 3 females; age range 20–27 years, mean BMI males 23.0 ± 1.9 kg/m^2^, females 22 ± 2.4 kg/m^2^;	Coffee: 5.1 ± 0.4 cups/day. 3.6 cups/day moka, 1.5 cups/day espresso; moka 40–50 mL/cup, espresso 25–35 mL/cup. Decaffeinated coffee intake was 24% of the total. Composition: n.d.	Coffee group: 5 cups coffee/day for 1 week Control group: No coffee consumption for 1 week	Parallel intervention	↑GSH			
Grubben et al. [42]	64 subjects (31 males and 33 females; mean age 43 ± 11 years, mean BMI 24.5 ± 0.5 kg/m^2^)	Coffee: blend of arabica and robusta beans. 39 g of ground coffee into a 1 L cafetière coffee-pot (1 L equals six cups) Composition: cafestol mean 34 ± 3 mg/L, kahweol mean 26 ± 1 mg/L	Coffee group: 1 L/day of unfiltered cafetière coffee for 2 weeks Control group: water, milk, tea chocolate drink or broth for 2 weeks	Randomized, controlled, crossover intervention	↑GSH (Colorectal mucosa and plasma)			
Kempf et al. [43]	47 subjects (11 males, 36 females), mean age 54.0 ± 9.0 years, mean BMI 29.2 ± 4.6 kg/m^2^	Coffee: Juhla Mokka branded coffee, made with participants’ coffee machines at home Composition: n.d.	Coffee group 1: 4 cups (150 mL per cup) of coffee for 4 weeks Coffee group 2: 8 cups (150 mL per cup) of coffee for 4 weeks Control group: n.d.	Single blind, three stages intervention		↓IsoPs		=3NT
Yukawa et al. [44]	11 healthy men, range age 21–31 years	Coffee: coffee freshly prepared by mixing 8 g of Arabica coffee with 150 mL water Composition: n.d.	Coffee group: 150 mL coffee (3 times per day for 1 week) Control group: mineral water for 1 week (amount not reported)	Baseline and post intervention		↓Susceptibility of LDL to oxidation ↓MDA		
Bakuradze et al. [45]	84 healthy subjects, mean age 25.6 ± 5.8 years, mean BMI 22.9 ± 1.9 kg/m^2^	Coffee 1: blend (SB) coffee: 100% Arabica Composition: 12.39 ± 0.1 mg/g caffeine, 19.31 ± 0.3 mg/g CGA, 0.39 ± 0.0 mg/g NMP and 6.27 ± 0.1 mg/g trigonelline Coffee 2: market blend (MB) coffee, obtained from equal portions of 4 Arabica and 1 Robusta commercially available regular coffee brands Composition: 12.8 ± 0.2 mg/g caffeine, 10.01 ± 0.3 mg/g CGA, 1.20 ± 0.0 NMP and 3.42 ± 0.2 mg/g trigonelline	Coffee group: 750 mL/day of MB or SB for 4 weeks Control group: n.d.	Randomized, non-controlled, cross-over intervention			↓SBs ↓FPG-sensitive sites	
Bakuradze et al. [46]	84 healthy men, range age 19–50 years, mean bw 80.9 ± 12.4 kg Coffee group: 42 men, mean BMI 24.9 ± 3.0 kg/m^2^ Control group: 42 men, mean BMI 24.4 ± 3.5 kg/m^2^	Coffee: Arabica coffee, freshly prepared in a pad machine Composition: 11.78 ± 0.42 mg/g caffeine, 10.18 ± 0.33 mg/g CGA, 1.10 ± 0.05 NMP and 3.82 ± 0.09 mg/g trigonelline	Coffee group: 750 mL/day of coffee for 4 weeks Control group: 750 mL/day of water for 4 weeks	Randomized, controlled, cross-over intervention			↓SBs	
Bichler et al. [47]	8 healthy non-smokers volunteers (age range 20–50 years, BMI n.d.)	Coffee: metal filtered coffee andpaper filtered coffee, both prepared with 50 g of ground coffee per liter Composition: n.d.	Coffee group: 600 mL coffee/day (200 mL metal filtered coffee and 400 mL paper filtered coffee) for 5 days Control group: No coffee consumption		=GPx		↓Endo and FPG-sensitive sites ↓H_2_O_2_- and Trp-P-2-induced DNA damage	
Cardin et al. [48]	37 patients with chronic hepatitis C (29 males, 8 females), mean age 58 ± 11 years, mean BMI 26 ± 5 kg/m^2^	Coffee: 100% Coffee Arabica prepared with an Italian-style coffee machine Composition: n.d.	Coffee group: 4 cups of coffee/day for 30 days Control group: no coffee consumption for 4 weeks	Randomized, controlled, cross-over intervention			↓8-OHdG	

Legend: 8-OHdG: 8-Hydroxydeoxyguanosine; AC: antioxidant capacity; BMI: body mass index; BPDE: (±)-anti-B[*a*]P-7,8-dihydrodiol-9,10-epoxide; bw: body weight; CAT: catalase; CGA: chlorogenic acids; ENDO III: endonuclease; FPG: formamidopyrimidine DNA glycosylase; FRAP: ferric-reducing antioxidant power; GAE: gallic acid equivalent; GPx: glutathione peroxidase; GSH: reduced glutathione; GSR: glutathione reductase; GSSG: oxidized glutathione; IsoPs: isoprostanes; MDA: malondialdehyde; NMP: *N*-methylpyridinium; NT: nytrotyrosine; ORAC: Oxygen radical absorbance capacity; OxLDL: oxidized LDL; SBs: strand breaks; SOD: superoxide dismutase; TAC: total antioxidant capacity; TAS: total antioxidant status; TEAC: total equivalent antioxidant capacity.
